# Generation of a biotinylatable Sox2 mouse model to identify Sox2 complexes in vivo

**DOI:** 10.1007/s11248-018-0058-1

**Published:** 2018-01-30

**Authors:** Kim Schilders, Evelien Eenjes, Gabriëla Edel, Anne Boerema de Munck, Marjon Buscop van Kempen, Jeroen Demmers, René Wijnen, Dick Tibboel, Robbert J. Rottier

**Affiliations:** 1 Department of Pediatric Surgery, Erasmus Medical Center-Sophia Children’s Hospital, Wytemaweg 80, 3015 CN Rotterdam, The Netherlands; 2 Netherlands Proteomics Center, Erasmus Medical Center, Rotterdam, The Netherlands; 3 Department of Cell Biology, Erasmus Medical Center, Rotterdam, The Netherlands

**Keywords:** Biotinylatable tag, Sox2, Knock-in, In vivo protein complexes

## Abstract

Sox2 is a Sry-box containing family member of related transcription factors sharing homology in their DNA binding domain. Sox2 is important during different stages of development, and previously we showed that Sox2 plays an important role in branching morphogenesis and epithelial cell differentiation in lung development. The transcriptional activity of Sox2 depends on its interaction with other proteins, leading to ‘complex-specific’ DNA binding and transcriptional regulation. In this study, we generated a mouse model containing a biotinylatable-tag targeted at the translational start site of the endogenous Sox2 gene (bioSox2). This tag was biotinylated by the bacterial birA protein and the resulting bioSox2 protein was used to identify associating partners of Sox2 at different phases of lung development in vivo (the Sox2 interactome). Homozygous bioSox2 mice are viable and fertile irrespective of the biotinylation of the bio tag, indicating that the bioSox2 gene is normally expressed and the protein is functional in all tissues. This suggests that partners of Sox2 are most likely able to associate with the bioSox2 protein. BioSox2 complexes were isolated with high affinity using streptavidin beads and analysed by MALDI-ToF mass spectrometry analysis. Several of the identified binding partners are already shown to have a respiratory phenotype. Two of these partners, Wdr5 and Tcf3, were validated to confirm their association in Sox2 complexes. This bioSox2 mouse model will be a valuable tool for isolating in vivo Sox2 complexes from different tissues.

## Introduction

Sox2 is a Sry-box containing family member of related transcription factors sharing homology in their DNA binding domain. Sox2 is highly conserved across species and is involved in several developmental processes (Pevny and Placzek 2005). Sox2 expression is temporally and spatially regulated during development and starts to be expressed at the morula-stage of development (Avilion et al. 2003; Liu et al. 2013). Expression becomes restricted to the inner cell mass of the blastocyst and continues in the epiblast, which will give rise to the embryo and germ cells (Avilion et al. 2003). Early during development, expression of Sox2 is restricted to the anterior ectoderm, from which the neuroectoderm and anterior surface ectoderm will arise (Papanayotou et al. 2008; Wood and Episkopou 1999). At later gestational ages, Sox2 is expressed in several tissues derived from the primitive foregut endoderm and post-natal it is present in the epithelium of foregut derived organs including the trachea and proximal lung epithelium (Avilion et al. 2003; Donner et al. 2007; Gontan et al. 2008; Ishii et al. 1998; Schlosser and Ahrens 2004; Taranova et al. 2006; Uchikawa et al. 2003). Importantly, together with Oct4 and Nanog, Sox2 is part of the core transcription factor network in embryonic stem cells, and Sox2 is one of the key factors required to induce pluripotent stem cells (iPSC) from in somatic cells (Avilion et al. 2003; Masui et al. 2007; Takahashi et al. 2007; Takahashi and Yamanaka 2006; Yamanaka 2012). Together with Oct4 and Klf4, Sox2 first co-occupies non-permissive chromatin and the subsequent activation of pluripotency-associated genes by Sox2 (Smith et al. 2016; Xu et al. 2016).

Sox2 for the induction and generation of Induced Pluripotent Stem Cells is warranted, especially since this transgenic line may be useful for understanding the transition from restricted cell types to reprogramming as pluripotent stem cell, and then the differentiation of these stem cells into specific cell types that retain Sox2 expression.

We and others have shown that Sox2 plays an important role in lung epithelial cell differentiation and branching morphogenesis (Gontan et al. 2008; Que et al. 2009; Tompkins et al. 2011). In iSox2SPC-rtTA mice lung, where Sox2 expression is induced in the epithelial cells of the developing airways, cystic lesions were observed (Gontan et al. 2008). The size of these cyst-like structures correlated with the timing and duration of ectopic Sox2 expression (Ochieng et al. 2014). The epithelium of these dilated airway structures had increasing numbers of basal cells and neuroendocrine cells (Gontan et al. 2008). In control lung Sox2 expression in the epithelial tip cells is inhibited by Fgf10 induced β-catenin signalling, which prevents these cells to differentiate in proximal epithelial cells (Domyan et al. 2011; Volckaert et al. 2013). Ectopic expression of Sox2 in these distal epithelial cells aberrantly induced these cells to differentiate into proximal cells, leading to the emergence of basal and neuroendocrine cells. Sox2 directly activated the ΔN Trp63 promoter, indicating that Sox2 is directly responsible for the emergence of basal cells (Ochieng et al. 2014).

The transcriptional activity of Sox2 depends on its interaction with other proteins, leading to ‘complex-specific’ DNA binding and transcriptional regulation (Kamachi and Kondoh 2013). Several studies have identified Sox2 associating partners in vitro using different kinds of cells (Ahmed et al. 2012; Boyer et al. 2005; Cox et al. 2010; Cox et al. 2013; Donner et al. 2007; Engelen et al. 2011; Fang et al. 2011; Inoue et al. 2007; Kamachi et al. 2001; Kondoh and Kamachi 2010). One of the partners identified in neural stem cells is Chd7. Sox2 and Chd7 cooperate to regulate genes involved in human syndromes that are genetically unrelated but do show a similarity in symptoms (Engelen et al. 2011). By performing large scale immunoprecipitations in stable FLAG/Sox2 transgene embryonic stem (ES) cells, several partners were identified including ES cell self-renewal factors and several lineage-specific transcription factors. Also, Xpo4 was identified as a Sox2 partner, functioning as a nuclear import receptor for Sox2 (Gontan et al. 2009).

Processes in which Sox2 is involved in lung development could be the influenced by specific interaction partners and therefore in vivo binding partners of Sox2 were identified in this study, using a mouse model containing a biotinylated Sox2 (bioSox2). The biotinylated Sox2 was efficiently isolated, including associating protein complexes due to the very high affinity of the biotin-streptavidin interaction, which is several magnitudes higher than antibody-antigen interactions (de Boer et al. 2003).

BioSox2 containing complexes were efficiently isolated from mouse embryonic day 18.5 lungs and brains using streptavidin and subsequently analysed by mass spectrometry. We identified a number of putative binding partners involved in lung development, such as Akap8, Ank3, Dkc1, Cavin (Ptrf) and Safb1.

## Materials and methods

### Generation of bioSox2/birA mice

To generate an N-terminal biotin-tagged Sox2 allele (bioSox2), Sox2 genomic DNA was isolated from library with 129 genomic DNA and a NheI—Asp718 fragment containing the Sox2 exon was used to generate the recombination construct used for targeting IB10 ES cells. Genomic DNA of individual clones was digested with EcoRI and screened with specific probes. The neomycin resistance gene was removed from positive clones by transiently expressing Cre in ES cells, and individual clones were genotyped and karyotyped before injection in blastocysts. Chimaeric mice were crossed and maintained on C57/bl6 background. BioSox2 mice were crossed with birA mice to biotinylate the biotag. Mice were kept under standard conditions and experiments were performed following guidelines of the ethics committee of the Erasmus Medical Center.

### Large scale tissue immunoprecipitations

Lungs and brains were isolated from bioSox2/birA and birA mice E18. Tissues were minced into small pieces and a single cell solution was prepared using a cell strainer. Immunoprecipitations were essentially done as previously published (Engelen et al. 2011; Gontan et al. 2009). Cells were lysed in cell lysis buffer (10 mM Hepes 7.6, 1.5 mM MgCl2, 10 mM KCl; add 0.5 mM DTT + protease inhibitors prior to use), followed by lysis of the nuclei in nuclei lysis buffer (20 mM Hepes pH 7.6, 20% glycerol, 420 mM NaCl, 1.5 mM MgCl2, 0.2 mM EDTA, add 0.5 mM DTT +1 × CEF prior to use). Nuclear extracts were diluted 1:1 in low-salt buffer (20 mM Hepes pH7.6, 20% glycerol, 1.5 mM MgCl_2_, 0.2 mM EDTA) and incubated with 40 µl Dynabeads^®^M-280 Streptavidin (Cat. No. 112.06 D, Invitrogen) for at least 1 h rotating at 4 °C in non-stick tubes. After washing with wash buffer (20 mM Hepes pH7.6, 20% glycerol, 100 mM KCl, 1.5 mM MgCl_2_, 0.2 mM EDTA, 0.02% NP-40; add 1 × CEF prior to use), the beads were resuspended in 40 µl sample buffer (60 mM Tris–HCl pH 6.8, 2% SDS, 0.02% bromophenol blue, 10% glycerol, 1% β-mercaptoethanol, 5 mM DTT) and heated for 10 min at 95 °C. Samples were loaded on pre-cast gel and sent to the department of Biochemistry for Matrix Assisted Laser Desorption/Ionization-Time of Flight Mass Spectrometry (MALDI-TOF MS) analysis.

### BioSox2 immunoprecipitation in transfected HEK cells

HEK cells were cultured in DMEM (Lonza, Verviers, Belgium) with 5% fetal calf serum and 1% penicillin–streptomycin under standard culture conditions. Cells were transfected with a N-terminal tagged bioTEV-Sox2 and birA expression constructs using Lipofectamine-2000 (Invitrogen) according to the manufacturer’s manual. Cells were harvested 24 h after transfection and nuclear extracts were prepared by lysing the cells with cell lysis buffer, followed by nuclei lysis buffer. Nuclear extracts were diluted 1:1 in low-salt buffer and incubated with Dynabeads^®^M-280 Streptavidin for at least 1 h rotating at 4 °C in non-stick tubes. After washing with wash buffer, the beads were resuspended in 40 µl sample buffer and heated for 10 min at 95 °C. Mock-transfected HEK cells were used as a control.

### Co-transfections and co-immunoprecipitations

HEK cells were transfected with either a myc-tagged TCF3 expression construct (Pereira et al. 2006), together with 2xFLAGbio-Sox2, or with a FLAG-tagged WDR5 construct (Lee and Skalnik 2005), together with myc-Sox2. X-tremeGENE HP DNA Transfection Reagent (Roche, Basel, Switzerland) was used for the transfection according to the manufacturer’s manual.^[6]^ Cells were harvested 24 h after transfection. Total cell extracts were prepared in 300 µl carin buffer (20 mM Tris pH8, 137 mM NaCl, 10 mM EDTA, 1% NP40, 10% glycerol) with Complete protease inhibitor (Roche, Basel, Switzerland). 50 µl was incubated for 2 h at 4 °C in 250 µl carin buffer with antibodies against myc (1668149, Roche) and FLAG (F1804), followed by 1 h incubation with protein G beads (Sigma-Aldrich, St. Louis, MO). After washing with carin buffer, the beads were resuspended in 20 µl sample buffer and heated for 10 min at 95 °C.

## Results

### Generation of bioSox2 mice

**Fig. 1:**
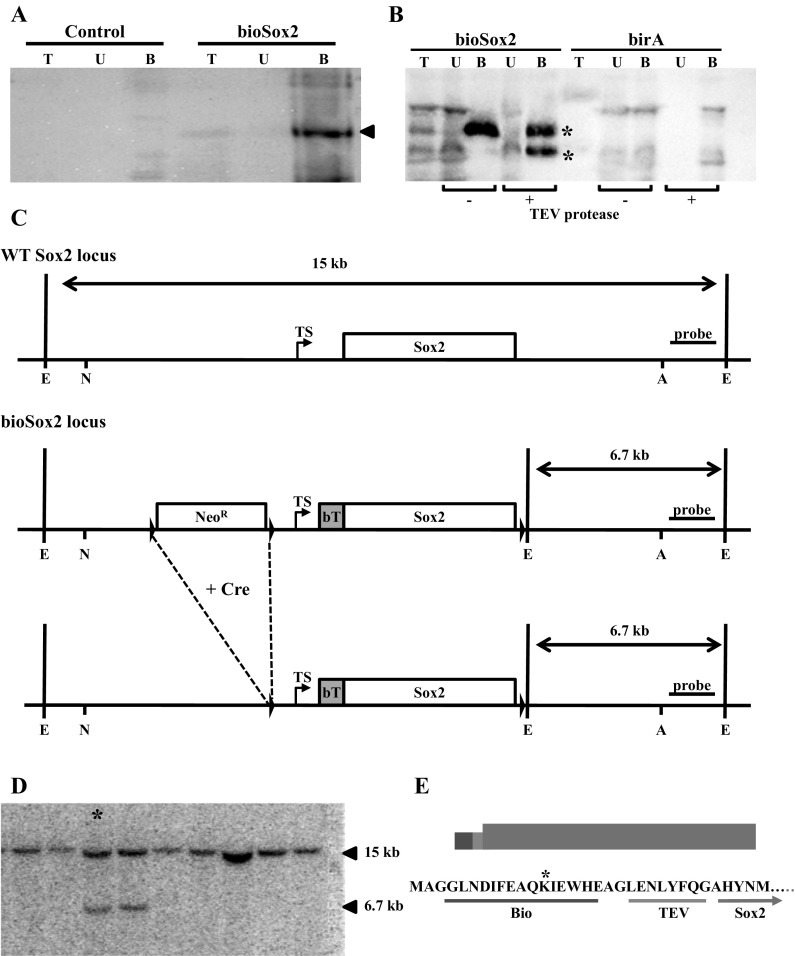
Development of biotin tagged Sox2 locus. **a** Nuclear extracts of transiently transfected HEK cells with control vector (control) or expression constructs for birA and bioSox2 (bioSox2) were incubated with streptavidin coupled dynabeads. Total input (*T*), unbound fraction (*U*) and the streptavidin bound fraction (*B*) analyzed on western blot using HRP coupled streptavidin. Arrow indicates the purified 40 kDa biotinylated Sox2 protein. **b** Identical experiment as in **a**, except that the purified samples were incubated with or without TEV protease. Although the bioSox2 was cleaved by the TEV, the protein remained attached to the beads. T is a fraction of the total input material before the beads were added to the extract. U represents the unbound fraction eluted from the beads after incubation with the TEV protease, whereas *B* represents the bound fraction, which was left on the beads after the TEV protease incubation. The *B* fraction was subsequently retrieved by boiling the beads. **c** Construct design to modify the Sox2 locus by homologous recombination. The Neomycin cassette was introduced upstream the transcriptional start site (*TS*), flanked by two loxP sites (arrow heads). The restriction sites used to isolate the fragment used to electroporate ES cells are indicated (N: NheI; A: Asp718), as well as the EcoRI sites (*E*) to analyze genomic DNA with the 3′ probe. **d** Representative Southern blot of ES genomic DNA digested with EcoRI and probed with the indicated 3′ probe, resulting in the wild type band at 15709 bp, and a mutant band 6771. **e** Schematic overview of the bioTEV-Sox2 protein and the N-terminal amino acids representing the translational start of the Sox2 protein (MA) followed by the Bio tag, a short hinge region (AGL) and the TEV protease cleavage recognition site (TEV). The asterisk indicates the lysine residue that is biotinylated by the BirA protein

Sox2 associating proteins have mostly been identified using a tagged Sox2 protein expressed in different cell lines (Cox et al. 2010, 2013; Engelen et al. 2011; Fang et al. 2011). In vitro cell culture models lack the microenvironment of cells in vivo, which could result in aberrant expression patterns and therefore a difference in partners in vitro and in vivo. Since the expression of Sox2 is temporally and spatially regulated, it is expected that there is also a dynamic change in interaction partners during the different stages of gestation. In order to isolate and identify in vivo Sox2 complexes with high specificity, we generated a mouse line expressing Sox2 with a small artificial peptide tag of seventeen amino acids (biotag) at the N-terminus. This biotag can be biotinylated in vivo by a biotin ligase protein (birA) and the protein can subsequently be isolated with high affinity using streptavidin (de Boer et al. 2003). As a proof of principle, the functionality of the biotinylated Sox2 protein was tested in vitro by co-expressing the N-terminal bio-tagged Sox2 with the bacterial birA biotin ligase. Previously, we showed that a N-terminal FLAG-tagged Sox2 protein was fully functional (Engelen et al. 2011; Gontan et al. 2009). The biotag was efficiently biotinylated by birA, as shown by western blot and by streptavidin specific precipitation of the biotinylated Sox2 from nuclear extracts (Fig. 1a). Moreover, the biotinylated Sox2 was able to bind to the Sox2 consensus binding site in vitro by an electrophoretic mobility shift assay (data not shown). We also tested the Tobacco Etch Virus (TEV) protease cleavage site, which was introduced after the biotag to facilitate the recovery of streptavidin precipitated bioSox2 from the magnetic streptavidin beads for subsequent analysis. BioSox2 and associating factors were purified from nuclear extracts of bioSox2/birA transfected HEK cells using magnetic streptavidin beads and precipitated proteins were released either by incubation with the TEV protease or by heating the beads for ten minutes. Western blot analysis showed that the TEV protease recognized and processed the bioSox2 protein, but unfortunately the cleaved Sox2 did not elute from the beads (Fig. 1b). Since the level of Sox2 is important, we decided to design a fragment of the Sox2 locus with the bio-tag to specifically target the endogenous locus by homologous recombination (Fig. 1c).

Therefore, a 11 kb NheI-Asp718 genomic fragment of 129 mouse DNA containing the Sox2 exon (− 4123 till + 7221, TS is position 1) was isolated. A fragment containing a Neomycin resistance gene (*Neo*) driven by the HSV-tk promoter and flanked by loxP sites was inserted in the SpeI site upstream the Sox2 exon (− 478), the site which has previously been used to construct Sox2 mutant mice (Taranova et al. 2006). With this fragment, an additional EcoRI site was created. Downstream of the Sox2 encoding region, in the SphI site (2445, counted from TS), a loxP site was introduced, also containing an EcoRI site. Finally, an oligonucleotide with the sequence coding for the 17 amino acid biotag and a TEV endoprotease cleavage site was inserted between the first and second amino acid encoding sequence of the Sox2 gene (+362). Thus, the translation initiates at the endogenous ATG and results in an in-frame insertion of the biotag leading to a fusion protein (bio-TEV-Sox2). The biotag can be biotinylated at a specific lysine residue by the bacterial birA ligase, as previously shown (de Boer et al. 2003). Homologous recombination was performed in 129/Ola derived IB10 ES cells, and subsequent isolated Neomycin resistant clones were analysed by Southern blotting (Fig. 1d). One of the ES clones that had the correct integration and the correct karyotype was subsequently used to transiently express Cre recombinase to remove the Neo cassette. After the removal of the cassette, the ES clone was injected into blastocysts and transferred to pseudopregnant females. The resulting mouse line contained the biotag in frame in the Sox2 coding sequence (see Fig. 1e), as well as loxP sites around the Sox2 exon, which may be used to genetically ablate the Sox2 gene. The mice that contained the correct targeted Sox2 locus (bioSox2 mice) were subsequently crossed with a mouse line ubiquitously expressing a HA-tagged birA from the ROSA26 locus to generate bioSox2/birA mice (Driegen et al. 2005).

**Fig. 2:**
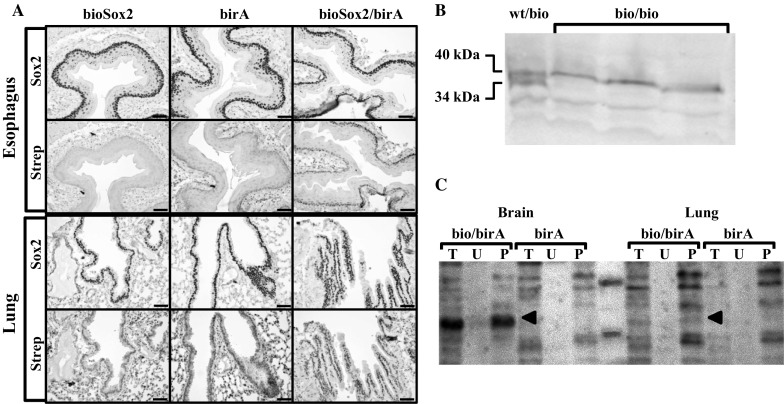
Functional analysis of the bioSox2 mouse. **a** Immunohistochemistry analysis of esophagus and lungs of adult bioSox2, birA and bioSox2/birA mice showing the expression of Sox2 using an antibody against Sox2 protein (Sox2). Using HRP coupled streptavidin (Strep) shows that the biotinylated Sox2 is expressed in the same cells as the normal Sox2. Moreover, biotinylation only occurs in the mice that carries the bioSox2 allele and the birA transgene. **b** Total protein extracts isolated from E 17.5 brains of heterozygous Sox2/bioSox2 mice (wt/bio) and homozygous bioSox2 mice (bio/bio) were analyzed by western blot analysis using a Sox2 antibody. This showed that the expression of the Sox2 (34 kDa) and bioSox2 (40 kDa) are comparable (lane wt/bio). **c** In vivo purification of bioSox2 from nuclear extracts of brain and lung tissue isolated at E18 from bioSox2/birA (bio/birA) and birA only (birA) mice using dynabeads. Arrowheads indicate the band representing the bioSox2, showing specific purification of the bioSox2 in the bioSox2/birA extracts. The western blot is labeled with streptavidine coupled HRP. Indicate lanes are total input (*T*), unbound fraction (*U*) and the purified fraction (*P*)

Homozygous bioSox2 mice, with or without the biotin ligated to the N-terminal tag, were born at Mendelian ratios, were viable and fertile. However, the targeted locus may interfere with the pattern of expression, thus we first compared the expression pattern of the biotinylated Sox2 with the endogenous, untagged Sox2. Several tissues were isolated from adult mice that either had the bioSox2 allele (bioSox2), carried the HA-BirA transgene (birA), or were double positive for the bioSox2 allele and the HA-BirA transgene (bioSox2/birA). Immunohistochemistry using an antibody against Sox2 or HRP-conjugated streptavidin showed that expression pattern of the bioSox2 in the esophagus and lung is similar to the endogenous Sox2 expression (Fig. 2a). the expression of the biotinylated Sox2 protein was identical to the control Sox2, as shown for lung and trachea (Fig. 2a). Next, we evaluated the level of transcription by isolating nuclear extracts of embryonic brains at gestational age 17.5. Subsequent western blot analysis showed that the bioSox2 (40 kDa) was equally expressed as the endogenous Sox2 (34 kDa; Fig. 2b). This provided evidence that the targeting itself did not interfere with the transcriptional and translational machinery.

Thus, the expression of the tagged bioSox2 was comparable to normal, untagged Sox2, and the bioSox2 was efficiently biotinylated in vivo by the birA protein. Collectively, this indicated that the *bioSox2* gene is normally expressed and the tagged protein is fully functional in all tissues. It also implies that all the partners of Sox2 are still able to associate with bioSox2 and fulfill their biological roles, since the absence of correct complex formation will lead to lethal phenotypes.

### BioSox2 affinity-purification

As a proof of principle, Sox2 complexes were isolated from fetal trachea and lung tissue isolated just prior to birth. At this phase of development, the epithelium of the trachea and upper airways consists of Sox2 positive cells, so total lungs were isolated at day 18.5 of gestation of bioSox2/HA-birA and HA-birA pups. Nuclear extracts were prepared and bioSox2 complexes were purified with streptavidin-coupled magnetic beads. The bioSox2 purification was performed in triplicate and the precipitation was first evaluated, indicating that the bioSox2 was efficiently purified from the bioSox2/birA mouse samples compared to the control birA only samples (Fig. 2c; arrowheads). It also showed that the bioSox2 protein was less prominently present in the lung samples, as expected, since Sox2 is expressed in a subset of epithelial cells.

**Fig. 3:**
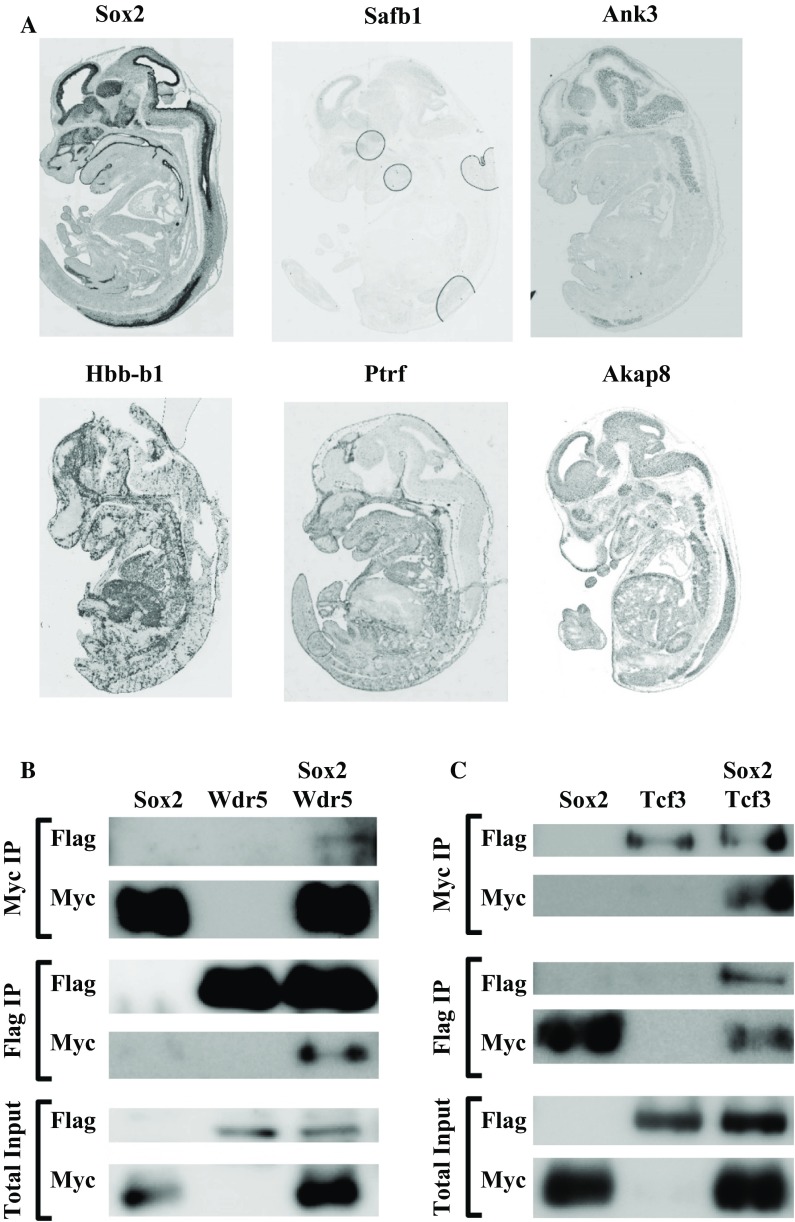
In vivo isolation of bioSox2 complexes. **a** Large scale purification of bioSox2 complexes from E18.5 lungs revealed several putative Sox2 associating proteins. The expression pattern of some of these partners is represented (genepaint). **b**, **c** Physical interaction between Wdr5 (**b**) and Tcf3 (**c**) with Sox2 was confirmed in co-immunoprecipitations. The myc antibody precipitated the myc-Sox2 (**b**) or myc-Tcf3 (**c**), and coprecipitated the FLAG tagged Wdr5 (**b**) or Sox2 (**c**). These interactions were confirmed by performing the reciprocal imunoprecipitations

The total precipitated proteins were separated on a polyacrylamide gel, which was stained with Coomassie Brilliant Blue and analyzed by MALDI-TOF MS. Comparing the three independent immunoprecipitations, 114 unique proteins were identified with a similar accession code and a mascot score above 80 that were enriched in the lung, while in the brain 28 unique proteins were found. Enrichment was determined by subtracting the number of unique proteins in the birA samples from the number of unique proteins in the bioSox2/birA samples. Aside from the identification of several potential binding partners that we previously described in epitope-tagged Sox2 pull down experiments performed with lysates from mouse embryonic stem cells and mouse neural stem cells (Engelen et al. 2011; Gontan et al. 2009), we found a number of potential partners that were linked to respiratory phenotypes in mice when ablated, including Akap8, Ank3, Dkc1, Cavin (Ptrf) and Safb1 (Fig. 3a).

### Wdr5 and Tcf3 are binding partners of Sox2

Putative binding partners were selected on the basis of their Mascot scores and the number of peptides retrieved in the mass spectrometry analysis. This set of proteins was subsequently analyzed for known cellular functions and their potential role in lung development, which resulted in a short list of putative Sox2 interacting proteins. To validate our results, we selected the WD repeat domain 5 (Wdr5) and Transcription factor 3 (Tcf3) proteins, since Wdr5 was described as a potential Sox2 binding partner in ES cells and we previously identified Tcf3 as a putative Sox2 binding protein in neural stem cells (Ang et al. 2011; Engelen et al. 2011).

Next, HEK cells were transiently transfected with expression constructs of a FLAG-tagged Sox2 and a myc-tagged Tcf3, or a myc-tagged Sox2 with a FLAG/HA-tagged Wdr5 to analyze their physical interaction. Extracts of transfected cells were incubated with the appropriate antibodies to immunoprecipitate the tagged Sox2 or Tcf3/Wdr5 and co-precipitated proteins were analyzed. We first analysed whether we could detect the interaction between Sox2 and one of its known partners, Wdr5. Immunoprecipitation of FLAG-Wdr5 with a FLAG antibody efficiently co-precipitated the myc-Sox2 protein as indicated by the myc positive signal (Fig. 3b). In the reverse experiment, immunoprecipitation of myc-Sox2 with a myc antibody showed co-precipitation the FLAG-Wdr5 protein with the FLAG antibody. Next, we analysed whether the putative partner, Tcf3, could also be co-precipitated with Sox2. Indeed, the FLAG-Sox2 efficiently associated with myc-Tcf3 as shown by the FLAG and myc specific precipitations (Fig. 3c). Thus, our data showed that aside from Wdr5, we identified Tcf3 as a specific partner of Sox2.

## Discussion

Several studies have identified Sox2 interaction partners in vitro using different approaches (Cox et al. 2010, 2013; Engelen et al. 2011; Fang et al. 2011). To gain more knowledge about the role of Sox2-partner complexes in vivo, we developed a mouse model expressing a biotinylatable Sox2 protein. As a proof of principle, we have efficiently purified the tagged Sox2 protein using streptavidin in a single-step approach using fetal lung.

The mass spectrometry data of the three large scale immunoprecipitations resulted in low scores of the bait, the bioSox2. This is partially due to the unfavourable distribution of the tryptic cleavage sites in the Sox2 protein and the presence of acidic amino acids within some of its peptides. Also, there is a low number of Sox2^+^ cells in the lung (~ 10%) compared to the brain where the bait-Sox2 score is much higher (not shown), showing that the assay itself works very efficiently. To obtain higher mascot scores and enrichment, we repeated the IP with a more protein as input. This resulted in a better enrichment of Sox2.

Tcf3 and Wdr5 were used to validate physical interaction with Sox2. Tcf3 was also previously identified as a potential Sox2 partner in a large scale purification assay in neural stem cells (Engelen et al. 2011). Tcf3 is involved in anterior–posterior axis induction during early embryonic development. Tcf3^−/−^ mice showed an expansion and duplication in the axial mesoderm (Merrill et al. 2004). Tcf3 is also involved in stem cell renewal, by inhibiting a subset of genes which results in repression of self-renewal (Yi et al. 2011). Tcf3 is expressed in skin epithelial cells and overexpression results in repression of epithelial cell differentiation (Merrill et al. 2001; Nguyen et al. 2006). Genetic ablation of the Tcf3-βcatenin interaction showed that this interaction is not required before gastrulation, but later during development in several crucial processes, such as vascular integrity and neural tube closure (Wu et al. 2012). It also showed that βcatenin relieves Tcf3 repression of Lef1, and subsequently activate Wnt target genes through its interaction with Lef1 (Wu et al. 2012). The antagonistic effect of Wnt signaling and Tcf3 expression was also confirmed in ES cells (Atlasi et al. 2013; Yi et al. 2011).

Wdr5 is involved in several processes including cell cycle progression and gene regulation. It interacts with Hdac3 under hypoxic conditions and then induces mesenchymal gene expression (Wu et al. 2011). As such, it plays a crucial role in hypoxia-induced epithelial-mesenchymal transition, which is important in processes as organ development and fibrosis. Wdr5 is also a direct target of Sry, as well as an interaction partner, and the Wdr5-Sry complex activates Sox9 and represses β-catenin expression in sex determination (Xu et al. 2012). In embryonic stem cells, Wdr5 is identified as a regulator of embryonic stem cell renewal. In these cells, Wdr5 is a direct binding partner of Oct4 and it has been shown that they have an overlap in gene regulatory functions. Immunoprecipitations done in the same study, also suggested interaction between Sox2 and Wdr5 (Ang et al. 2011). Co-immunoprecipitations that we performed validated this interaction. Wdr5 was also enriched in our first large scale bioSox2 immunoprecipitation in both the lung and trachea, suggesting that the Wdr5-Sox2 protein complex can be involved in lung development.

Several of the potential partners identified in the lung tissue IP can be linked to a respiratory phenotype in mice. Akap 8 (AKAP95), a member of the A-kinase anchor protein family, is a scaffold protein. Akap8 is together with fidgetin critical for palatogenesis in mice. Mice with only Akap8 mutations do not show any abnormalities, but mice with both Akap8 and fidgetin deficiencies show symptoms of respiratory distress and die due to cleft palate (Yang et al. 2006). Ankyrin 3 (Ank3/Ankyrin G) is a protein that is linked to integral membrane proteins. In bronchial epithelial cells, Ankyrin G is necessary for the biogenesis and preservation of the lateral membrane (Kizhatil and Bennett 2004; Kizhatil et al. 2007). Ank3 mutant mice show abnormal bronchus epithelium morphology (Jenkins et al. 2013). Dyskerin (DKC1) is linked to dyskeratosis congenital, which is characterized by premature aging and a higher tumor susceptibility. Patients suffering from this disease also display interstitial lung fibrosis. Hypomorphic Dkc1 mutant mice show lung abnormalities, including abnormal morphology of the pulmonary parenchyma, alveolus and alveolus wall, lung inflammation and pulmonary interstitial fibrosis (Ruggero et al. 2003). Cavin1 (Ptrf) is a protein that is involved in the regulation of rRNA transcription. Deletion of Cavin1 results in loss of caveolae in the lung, increased density of lung tissue and elevated pulmonary arterial pressure (Sward et al. 2013). Deletion of Cavin2 results in loss of endothelial caveolae in lung tissue (Hansen et al. 2013). Ptrf abnormal lung morphology and abnormal vasculature morphology. Scaffold attachment factor B1 (Safb1) is involved in development, growth and reproduction. abnormal lung alveolus development and Safb1^−/−^ mice show defects in lung maturation resulting in abnormal development of the alveoli (Ivanova et al. 2005). Similarities in the expression pattern of Sox2 and these potential partners at E14.5 can be found in supplementary data 1 (Genepaint, Visel et al. 2004).

In conclusion, we generated a mouse model containing a biotinylatable-tag targeted at the translational start site of the endogenous Sox2 gene. BioSox2 containing complexes can efficiently be isolated from various Sox2 expressing tissues and cell types using the mouse model.
